# From the skin to the thyroid: immunological mechanisms and therapeutic implications of the comorbidity between chronic spontaneous urticaria and autoimmune thyroid disease

**DOI:** 10.3389/fimmu.2026.1916978

**Published:** 2026-10-27

**Authors:** Qianqian Chen, Ting Yang, Xiaodan Hu, Rukang Chen

**Affiliations:** 1 Guang dong Provincial Hospital of Chinese Medicine, Guizhou Hospital, Guizhou, China; 2 The Traditional Chinese Medicine Hospital Of Longquanyi, Chengdu, China; 3 SCATCM Key Laboratory of Traditional Chinese Medicine Health Products Research andClinical Evaluation (Yibin Traditional Chinese Medicine Hospital); Yibin Clinical Research Center for Traditional Chinese Medicine Health Products, Yibin, China; 4 Department of Dermatology, The Second Affiliated Hospital of Zhejiang Chinese Medical University, Zhejiang, China

**Keywords:** autoimmune thyroid disease, autoimmunity, chronic spontaneous urticaria, Hashimoto’s thyroiditis, Thyroid autoantibodies

## Abstract

Chronic spontaneous urticaria (CSU) is the most common subtype of chronic urticaria, characterized by recurrent wheals and pruritus without identifiable triggers. CSU is a complex autoimmune skin disease, and patients with type I and type IIb autoimmune subtypes frequently have comorbid autoimmune thyroid diseases (AITD), such as Hashimoto’s thyroiditis (HT), suggesting shared immunological mechanisms between the two conditions. This article systematically reviews the epidemiological characteristics, multidimensional pathogenesis, and advances in clinical diagnosis and treatment strategies for CSU–AITD comorbidity. We focus on the roles of thyroid autoantibodies, immunoglobulin E (IgE)-mediated immune responses, and the complement system in disease progression, highlight the controversial mechanisms linking the two conditions and their therapeutic implications, summarize current treatment strategies targeting the comorbidity, and explore whether thyroid antibodies represent a causative factor or merely an epiphenomenon.

## 1 Introduction

Chronic spontaneous urticaria (CSU) is a common skin disorder characterized by recurrent wheals and/or angioedema without identifiable triggers, with a global prevalence of approximately 1% (1–3). Genetic studies have revealed that the pathogenesis of CSU is complex and shares genetic overlap with multiple immune-mediated diseases; the relevant genes are predominantly involved in immune regulation and inflammatory pathways, supporting the central role of autoimmunity in the pathogenesis of CSU (4–7).

From an immunological perspective, two major autoimmune subtypes have been identified in CSU: type I (autoallergic type, mediated by immunoglobulin E (IgE) autoantibodies) and type IIb (mediated by immunoglobulin G (IgG) autoantibodies). These subtypes differ in clinical manifestations and treatment responses, and may overlap in some patients, further reflecting the complexity of the immune mechanisms underlying CSU (2, 8–11).

The autoimmune nature of CSU is also reflected in its comorbidity profile, particularly in the strong association with autoimmune thyroid diseases (AITD). Studies have shown that the proportion of CSU patients with comorbid AITD is significantly higher than that in the general population (12, 13). AITD is not only the most common autoimmune comorbidity of CSU, but may also be deeply involved in its pathogenesis and progression. In patients with type IIb CSU, low total IgE levels combined with positive thyroid autoantibodies may serve as a surrogate marker for disease activity, treatment response, and prognosis (8, 10).

Thus, the comorbidity of CSU and AITD involves multiple processes, including genetic susceptibility, autoantibody production, immune cell dysregulation, and the release of inflammatory mediators, reflecting a profound overlap in immune dysregulation between the two conditions. This raises important questions: are thyroid antibodies a causative factor or merely an epiphenomenon? Does correcting thyroid dysfunction affect the course of CSU? Elucidating these mechanisms will not only help reveal the nature of this comorbidity, but also promote the development of individualized treatment strategies, providing evidence-based support for early identification and comprehensive management.

## 2 Epidemiological features of the comorbidity between CSU and AITD

Studies suggest that patients with CSU have an increased risk of autoimmune comorbidities; clinically, these may include AITD, vitiligo, rheumatoid arthritis (RA), type 1 diabetes (T1DM), and celiac disease (CD), among which AITD is the most prominent (14). Systematic reviews have shown that the positivity rate of thyroid autoantibodies, such as anti-thyroid peroxidase (anti-TPO) antibodies, in CSU is ≥10%. Among thyroid dysfunctions, hypothyroidism caused by Hashimoto’s thyroiditis (HT) is the most common, while hyperthyroidism is relatively rare and mainly associated with Graves’ disease (GD). The reported frequencies of the other comorbidities mentioned above in CSU are generally below 10%, suggesting that thyroid autoimmunity holds particular importance within the comorbidity spectrum of CSU (15).

The reported positivity rates vary across studies due to differences in the enrolled populations (primary care vs. specialty settings) and detection methods. A large study including 12,778 patients with CSU showed that the prevalence of hypothyroidism was 9.8%, much higher than that of hyperthyroidism. Among 3,662 patients who underwent serological testing, the positivity rates of anti-TPO and anti-thyroglobulin (anti-Tg) antibodies were 8.1% and 1.9%, respectively. The prevalence of hypothyroidism and the positivity rates of thyroid antibodies were both higher in females, and higher in adult patients than in children (16). A prospective cohort study further showed that CSU patients with concomitant hypothyroidism experience a longer disease course and have a significantly reduced likelihood of achieving clinical remission, with a hazard ratio (HR) of 0.425 (95% CI: 0.290–0.621) (17).

Thyroid autoantibodies are important serological evidence for the comorbidity association. Studies have demonstrated that up to 46% of CSU patients test positive for anti-TPO antibodies, a rate significantly higher than that of healthy controls (6.6%), indicating the presence of systemic autoimmune activation (18). Another study reported that the positivity rates of anti-TPO and anti-Tg antibodies among CSU patients were as high as 51.2% and 41.9%, respectively, which may be related to variations in the enrolled populations and detection methods (13). Studies suggest that different antibody types may point to distinct clinical subtypes and prognoses: anti-TPO IgG positivity is associated with an increased risk of angioedema, whereas anti-TPO IgE positivity may predict a better response to antihistamine therapy (19). In addition, thyroid ultrasound examinations have revealed that the incidence of structural heterogeneity (such as diffuse hypoechogenicity) and nodular changes of the thyroid gland is also significantly increased in CSU patients (13), suggesting that the autoimmune burden in CSU patients is not only reflected in antibody levels and functional abnormalities but may also directly lead to structural changes in the target organ.

In summary, there is a significant epidemiological association between CSU and AITD, with HT (often manifesting as hypothyroidism) being the most prominent. The high positivity rate of thyroid autoantibodies (especially anti-TPO antibodies) serves as an important marker of this association, and their different isotypes (IgG/IgE) have potential value for clinical phenotyping and prognostic prediction.

## 3 Shared autoimmune profiling of CSU and AITD

### 3.1 Autoantibodies and mast cell activation

At present, the mechanisms underlying the association between CSU and AITD have not been fully elucidated. Studies suggest that abnormal autoimmune responses play an important role in the comorbid progression of these two conditions. Thyroid autoantibodies, such as anti-TPO and anti-Tg antibodies, are hallmark antibodies of AITD, and their positivity rates are also significantly elevated in patients with CSU. Bioinformatics simulation studies have suggested that TPO and Tg proteins contain multiple linear and conformational epitopes that can be recognized by IgE and IgG autoantibodies. These cross-reactive antigenic epitopes may mediate autoimmune cross-reactivity, thereby jointly contributing to the pathogenesis of CSU and AITD. Moreover, patients with type I (autoallergic) CSU show a significantly higher prevalence of AITD and thyroid autoantibody positivity. In these patients, IgE autoantibodies targeting thyroid antigens (such as TPO) have been detected, suggesting their involvement in the pathogenesis of CSU (20, 21).

Anti-TPO IgE antibodies can cross-link the high-affinity IgE receptor (FcϵRI) on the surface of mast cells (MCs) and basophils via a type I hypersensitivity reaction, potentially triggering cell activation and degranulation, thereby inducing wheals or angioedema (22). Clinical data show that CSU patients with elevated anti-TPO IgE antibodies have a significantly higher prevalence of AITD than healthy controls (23). Although the IgE–TPO pathway has attracted considerable attention, antibody levels vary widely across studies, suggesting that IgE-mediated autoimmune responses are highly complex and heterogeneous (24).

In patients with type IIb (autoantibody-positive) CSU, IgG autoantibodies against IgE and its high-affinity receptor FcϵRI can be detected (2, 20). These autoantibodies not only participate in the initiation and maintenance of chronic inflammation through multiple mechanisms, but also serve as an important link between CSU and AITD. Patients with type IIb CSU tend to have low total IgE levels, yet often present with markedly elevated anti-TPO IgG antibodies; this serological combination is associated with poor response to omalizumab and may serve as a surrogate marker rather than a diagnostic criterion (8, 10). Clinical data show that patients with type IIb CSU are predominantly female and often have comorbid autoimmune diseases, including AITD and vitiligo (20, 25). Meanwhile, the potential association between anti-TPO IgG—which is not considered directly pathogenic—and MCs remains to be elucidated.

Beyond autoantibody-mediated activation, MCs may also participate in the comorbidity through direct interaction with the thyroid hormone system. Studies suggest that MCs express the thyroid-stimulating hormone (TSH) receptor and possess the capacity to store and metabolize thyroid hormones (26). Under inflammatory conditions, levels of thyroid hormone metabolites such as 3,5-diiodothyronine (3,5-T2), 3-iodothyronamine (T1AM), and 3-iodothyroacetic acid (TA1) are elevated; these metabolites may participate in regulating pruritus and pain by modulating histamine release and neural sensitivity.

These findings further deepen the understanding of the comorbidity mechanisms between CSU and AITD, suggesting a cross-regulatory role among MCs, the TSH receptor, and thyroid hormone metabolism. They also indicate that MCs may serve as a critical link mediating the skin–thyroid organ comorbidity, providing an important theoretical basis and research direction for identifying novel diagnostic markers and exploring precise therapeutic targets for CSU comorbid with AITD. Notably, type I CSU responds well to omalizumab and conventional treatments (27), whereas type IIb CSU typically presents with more refractory clinical symptoms and stronger treatment resistance, often showing suboptimal responses to conventional medications; however, this still requires further prospective validation (**Figure 1**).

**Figure 1:**
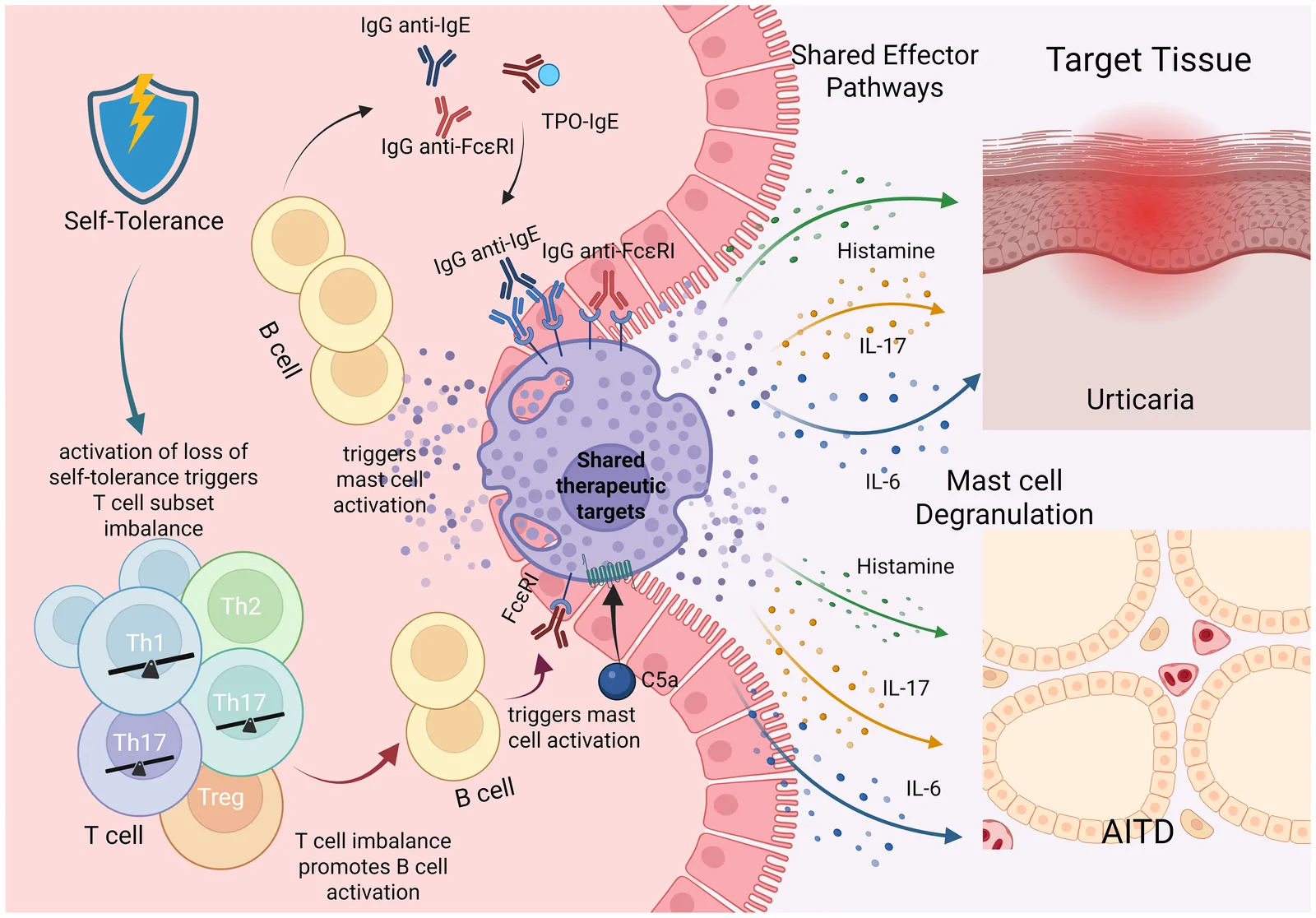
Shared immunopathological network in CSU and AITD. (Created with BioRender.com). This figure illustrates the core immune dysregulation common to both diseases. Central to this network is the breakdown of self-tolerance, leading to T-cell subset imbalance (Th1/Th2, Th17/Treg) and B-cell activation. This drives the production of autoreactive antibodies (e.g., anti-FcϵRI, anti-IgE, and anti-TPO IgG/IgE). These antibodies, together with complement activation (C5a), activate mast cells, the key effector cells. The resultant release of mediators (e.g., histamine, IL-17, IL-6, and thyroid hormone metabolites) contributes to both cutaneous symptoms (urticaria) and thyroid tissue damage in AITD. The overlapping pathways highlighted in the center represent shared therapeutic targets. CSU, chronic spontaneous urticaria; AITD, autoimmune thyroid disease; Th1, T helper type 1; Th2, T helper type 2; Th17, T helper type 17; Treg, regulatory T cell; anti-FcϵRI, anti-high-affinity IgE receptor antibodies; anti-IgE, anti-immunoglobulin E antibodies; anti-TPO, anti-thyroid peroxidase antibodies; IgG, immunoglobulin G; IgE, immunoglobulin E; C5a, complement component 5a; IL-17, interleukin-17; IL-6, interleukin-6.

### 3.2 T/B cell imbalance and immune tolerance deficiency

CSU and AITD share highly similar features of cellular immune dysregulation, mainly manifested as abnormal upregulation of T helper type 17 (Th17) cells, reduced numbers and functional defects of regulatory T cells (Tregs), which represent important common immunological characteristics of CSU and AITD, especially HT. In the immune dysregulation of CSU, in addition to the classical Th1/Th2 cell shift (28, 29), the role of Th17/Treg imbalance in disease progression has become increasingly important. In patients with CSU, the proportion of Th17 cells in peripheral blood and skin lesions is significantly increased, with massive release of pro-inflammatory cytokines such as Interleukin-17 (IL-17) and Interleukin-21 (IL-21); meanwhile, the number of Tregs is reduced, FoxP3 expression is downregulated, accompanied by insufficient secretion of anti-inflammatory cytokines such as Interleukin-35 (IL-35). This results in impaired immune tolerance, which ultimately induces persistent skin inflammation, increased vascular permeability, and recurrent episodes of chronic urticaria. Moreover, the degree of Th17/Treg imbalance is closely correlated with the severity of CSU (29, 30).

Highly consistent with this, excessive Th17 activation and Treg functional deficiency are major pathological features of AITD, a feature particularly typical in HT. In patients with HT, Th17 cells in thyroid tissue and peripheral blood exhibit significant proliferation and activation, secreting large amounts of IL-17, which directly mediates local inflammatory infiltration of the thyroid, destruction of follicular architecture, and tissue damage (31). Meanwhile, HT patients show reduced Treg numbers and decreased FoxP3 mRNA expression, and Tregs can convert into pathogenic Th1 and Th17 cells, significantly impairing autoimmune tolerance and driving chronic autoimmune injury of the thyroid (32, 33). Patients with GD also exhibit elevated Th17 cells, reduced Treg numbers, and functional Treg defects; these alterations are closely correlated with the activity of thyrotropin receptor antibodies (TRAb) and thyroid-stimulating antibodies (TSAb), further suggesting that Th17/Treg imbalance is a common mechanism underlying the pathogenesis of AITD (34, 35).

CSU and AITD share similar B-cell immune dysregulation mechanisms. In the pathogenesis of CSU, autoantigens such as TPO and Interleukin-24 (IL-24) can induce abnormal activation of autoreactive B cells, which proliferate and differentiate into plasma cells that continuously produce pathogenic autoantibodies such as IgE and IgG. When the body is re-exposed to the same antigens, effector cells are triggered to activate and release large amounts of inflammatory mediators, ultimately inducing typical urticarial manifestations such as wheals and pruritus (36). Meanwhile, the dysregulation of the cytokine network further exacerbates abnormal B-cell activation: decreased IL-21 levels can weaken the inhibitory effect on B-cell proliferation, and inflammatory cytokines such as Interleukin-4 (IL-4) and Interleukin-6 (IL-6) secreted by basophils can also enhance B-cell survival and proliferation, continuously amplifying humoral immune dysregulation and driving the chronic persistence of CSU (37, 38). This pattern also holds true in AITD, as represented by HT. In affected patients, abnormally activated autoreactive B cells can differentiate into plasma cells that secrete large quantities of thyroid pathogenic autoantibodies such as anti-TPO and anti-Tg, while also releasing pro-inflammatory cytokines that directly aggravate local inflammatory infiltration and follicular damage in the thyroid. Meanwhile, B cells can activate naive CD4^+^ T cells through antigen presentation, and the activated T cells in turn promote B-cell proliferation and differentiation, forming a positive feedback loop between B cells and T cells that continuously amplifies autoimmune dysregulation (39).

The pathogenic features of B cells in GD are even more typical. Specific antibodies such as TRAb and TSAb produced by activated autoreactive B cells are important pathogenic factors that induce hyperthyroidism and structural abnormalities of the thyroid. Moreover, the proportions of pathogenic B-cell subsets (IgD^-^CD27^-^ double-negative B cells and naive B cells) are significantly elevated in patients with GD and are positively correlated with autoantibody titers, suggesting that B-cell overactivation may be involved in the pathogenesis of AITD (40, 41). In addition, insufficient numbers and functional defects of regulatory B cells (Bregs) represent an important shared component of immune dysregulation in CSU and AITD, and are also a key contributor to the breakdown of immune tolerance. Bregs maintain autoimmune homeostasis primarily by secreting anti-inflammatory cytokines such as IL-10 and IL-35, thereby suppressing excessive pro-inflammatory responses mediated by Th1 and Th17 cells. Patients with CSU exhibit significant Breg dysfunction, rendering these cells unable to effectively suppress aberrantly activated B-cell and T-cell immune responses. This deficiency is even more pronounced in patients with AITD. Patients with HT exhibit reduced Breg numbers and weakened immunosuppressive function. In patients with GD, important Breg subsets such as CD19^+^CD1d^high^CD5^+^ and CD19^+^IL-10^+^ B cells are significantly decreased, and their levels are negatively correlated with pathogenic autoantibody titers, markedly weakening the counterbalance against autoimmune responses (35, 42) (**Figure 2**).

**Figure 2:**
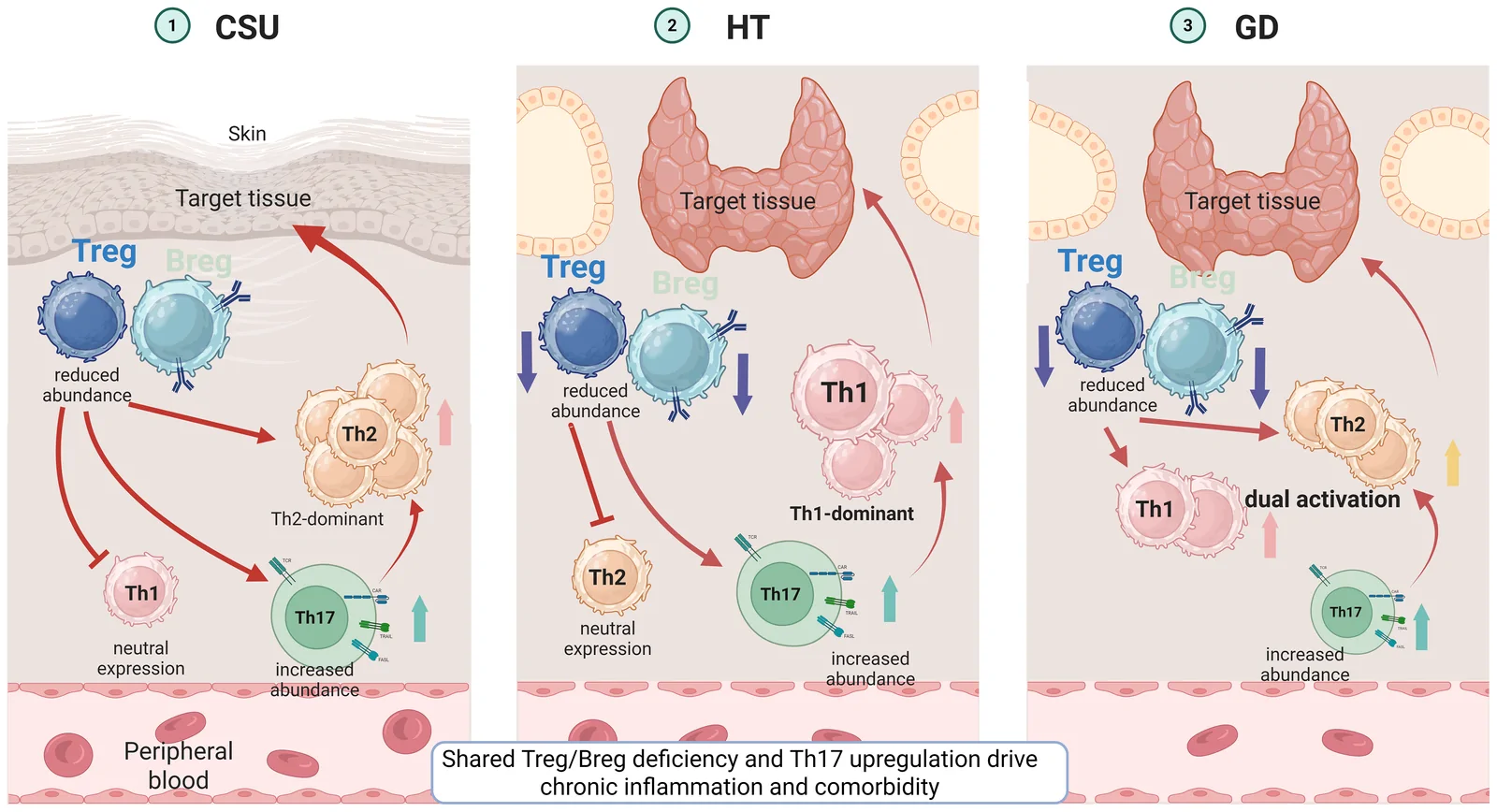
Comparative analysis of T- and B-Cell subset dysregulation in CSU, HT, and GD. (Created with BioRender.com). This schematic compares the status of key lymphocyte subsets. A common pattern of reduced numbers and impaired function of regulatory T and B cells (Treg/Breg) is observed across all three conditions. CSU is characterized by a Th2-dominant profile, whereas HT shows a Th1-dominant profile; GD exhibits both Th1 and Th2 activation. Th17 cells are consistently upregulated in all three conditions, contributing to chronic inflammation. This shared dysregulation forms the immunological basis for their high comorbidity. CSU, chronic spontaneous urticaria; HT, Hashimoto’s thyroiditis; GD, Graves’ disease; Th1, T helper type 1; Th2, T helper type 2; Th17, T helper type 17; Treg, regulatory T cell; Breg, regulatory B cell.

### 3.3 Cytokine- and complement-mediated inflammatory amplification

At the cytokine level, both CSU and AITD exhibit abnormal expression of multiple autoimmune-related inflammatory cytokines, among which the coordinated elevation of IL-17 is the most typical. As the major effector molecule of Th17 cells, IL-17 is significantly overexpressed in both diseases. In CSU, IL-17 can amplify Interferon-gamma (IFN-γ) and IL-4 signaling pathways, activate keratinocytes, and mediate T cell-related immune responses, while also inducing vasodilation, increased vascular permeability, and mast cell activation, directly promoting the development of wheals and pruritus (43). In AITD, IL-17 is abundantly expressed in thyroid tissue and areas of lymphocyte infiltration, participating in autoimmune thyroid damage. IL-6 also plays an important role in the comorbidity mechanism. In CSU, IL-6 can be secreted by T cells, macrophages, and basophils; it can not only enhance the proliferation, maturation, and reactivity of MCs (44, 45), but also promote B cell differentiation and autoantibody production, thereby driving disease progression (38). In AITD, IL-6 is likewise involved in humoral immune activation, promoting B cells and plasma cells to produce anti-thyroid autoantibodies and exacerbating local inflammation. Meanwhile, IL-17 and IL-6 may synergistically link T cell imbalance with mast cell activation, serving as one of the important nodes in the inflammatory network. Interferon lambda 1 (IFN-λ1; also known as IL-29), a member of the type III interferon family, is another important molecule connecting the two. *In vitro*, this molecule can reduce Th2 cytokine secretion and shift the immune response toward a Th1 profile. Serum IL-29 levels are significantly elevated in HT patients, which is consistent with the predominantly Th1 immune characteristics of HT (46). IFN-λ1 is also highly expressed in the peripheral blood and lesional skin of CSU patients, and may exacerbate the Th1/Th2 imbalance through the aforementioned Th1-skewing effect, suggesting that it occupies a common pro-inflammatory position in autoimmune skin and thyroid disorders (47).

In addition, CSU and AITD share the upregulation of multiple inflammatory cytokines such as tumor necrosis factor -alpha (TNF-α) and IL-23, which together constitute a highly overlapping and mutually amplifying cytokine network that drives the persistence of chronic inflammation. This high degree of similarity in cytokine profiles suggests that the two diseases may share some inflammatory pathways, although a common pathogenic mechanism cannot be inferred from this alone. Nevertheless, this similarity still provides important clues for understanding the association between the two conditions. Notably, AITD (especially HT) is dominated by a Th1-type immune response, with inflammation concentrated in thyroid tissue. In contrast, the immune polarization pattern of CSU varies by endotype—type I is characterized by a Th2-type response, while type IIb involves more Th1/Th17-related pathways—yet inflammatory cells infiltrate the lesional skin in both cases. This differential pattern of immune imbalance may reflect the heterogeneity of the immune microenvironment across different target organs and disease endotypes against the backdrop of a shared systemic autoimmune predisposition, and it also provides clues for understanding the two conditions as distinct manifestations within the spectrum of immune-related diseases.

The complement system, as an important component of innate immunity, also plays a significant role in the immunopathological processes of CSU and AITD. Abnormal complement activation can not only amplify local inflammation, but also recruit and activate neutrophils through its cleavage product C5a, aggravating cutaneous vascular damage and inflammatory responses. A study involving 30 CSU patients showed that more than half of the patients had C4d deposition in lesional skin, suggesting that immune complex-mediated activation of the classical complement pathway is involved in the pathogenesis of CSU. However, given the small sample size (n = 30), the findings should be interpreted with caution. Meanwhile, peripheral blood C5a levels were significantly elevated in these patients, further supporting the potential involvement of complement in mediating inflammation in CSU (48). Abnormal activation of the complement system is likewise involved in the immunopathological processes of AITD. Studies have found that membrane attack complex deposits are present in the thyroid tissue of AITD patients, and that thyroid tissue from HT patients is characterized by increased C1q deposition, with significantly elevated serum levels of complement components such as C4d (49). These findings suggest that complement activation can mediate thyroid tissue destruction and chronic inflammation by promoting autoantibody production and immune complex deposition. Thus, abnormal activation of the complement system may represent another important immunological link connecting CSU and AITD, as both conditions involve complement-mediated tissue damage and amplification of inflammation, providing further clues for understanding the immunopathological association between the two diseases.

## 4 Integrated strategies for diagnosis and assessment

### 4.1 Thyroid-related testing in CSU patients

CSU is a chronic inflammatory skin disease centered on autoimmunity, often significantly impairing patients’ quality of life through intense pruritus and a protracted disease course. AITD is the most common autoimmune comorbidity of CSU, with HT being the most prominent. The prevalence of hypothyroidism in patients with CSU is 9.8%, substantially higher than that of hyperthyroidism (16); hypothyroidism in CSU is most often attributable to HT, although it can also result from non-autoimmune causes. In addition, the positivity rates of anti-TPO antibodies in patients with CSU are markedly elevated, reported as 46% and 51.2% in two studies, respectively (13, 18). Furthermore, thyroid dysfunction is more common in females than in males, and in adults than in children (15).

The high degree of clinical overlap between the two conditions supports the importance of thyroid function and immune status screening in the management of CSU. Routine screening of thyroid immune status is of particular clinical significance for patients with moderate-to-severe CSU, a long disease duration, or resistance to conventional treatment. Positivity for thyroid antibodies not only indicates a potential risk of thyroid dysfunction but may also influence the clinical presentation, disease severity, and treatment response of urticaria.

Thyroid function testing is the foundation for evaluating thyroid status in patients with CSU, with the main indicators including thyroid-stimulating hormone (TSH), free triiodothyronine (FT3), and free thyroxine (FT4). TSH is the first-line marker for screening thyroid function; elevated TSH levels suggest hypothyroidism, whereas decreased levels indicate hyperthyroidism. FT3 and FT4 directly reflect the levels of biologically available thyroid hormones and serve as important parameters for assessing metabolic status and thyroid function. Assessment of thyroid autoantibodies is a key indicator for evaluating systemic autoimmune activation. Positivity for anti-TPO and anti-Tg antibodies is an important serological marker of AITD, particularly HT. Although the specific mechanisms by which these antibodies influence the clinical presentation and treatment response of CSU remain to be elucidated (50), their positive results are of significant reference value for identifying comorbidities, assessing disease activity, and predicting prognosis.

In summary, thyroid function tests (TSH, FT3, FT4) and thyroid autoantibody tests (anti-TPO, anti-Tg) are important tools for the diagnosis and evaluation of thyroid disease. All patients with CSU, especially those with refractory disease, should undergo routine screening of thyroid immune status to enable early detection and intervention. Conversely, patients with AITD should be monitored for skin symptoms to facilitate early identification of CSU. In clinical practice, test results should be interpreted in conjunction with symptoms and signs for a comprehensive assessment.

### 4.2 Potential biomarkers for CSU comorbid with AITD

Building upon the aforementioned routine tests, the identification of more specific biomarkers is particularly important for achieving more precise subtyping and prognostic assessment. Studies have shown that the combination of a positive autologous serum skin test (ASST), low serum total IgE levels, and anti-TPO IgG positivity may indicate type IIb autoimmune CSU (51). Such patients often present with high disease activity and poor response to conventional treatment, suggesting the need for more aggressive and individualized therapeutic strategies. In particular, ASST-positive patients typically exhibit higher disease activity and more severe clinical symptoms, and ASST positivity is closely associated with thyroid autoantibody positivity, such as anti-TPO antibodies (52), making it an important indicator for CSU comorbid with AITD.

Aberrant activation of the complement system may contribute to the amplification of inflammation in CSU–AITD comorbidity. Studies have shown that in patients with type IIb CSU, complement components (such as C4d) are often deposited in skin lesions, and serum C5a levels are elevated (48). Detection of complement components and fragments (such as C3, C4, C1q, C5a) may have auxiliary value in assessing the activity of type IIb autoimmune CSU. However, the dynamic relationship between complement levels, disease activity, and autoantibody titers remains unclear, and the lack of standardized testing limits its clinical application; thus, complement assessment is still at an exploratory stage.

Overall, biomarkers such as ASST, serum total IgE, and complement components, combined with conventional thyroid function and antibody testing, constitute a multidimensional toolkit for evaluating CSU–AITD comorbidity. At present, this biomarker combination remains an auxiliary tool and is not yet sufficient to independently guide treatment decisions. In the future, integrating these markers into predictive models may enable earlier and more accurate identification of high-risk comorbid patients and guide individualized treatment selection, thereby achieving a transition from “comorbidity diagnosis” to “precision management.”

## 5 Therapeutic implications and future directions

### 5.1 Therapeutic value of correcting thyroid function

Levothyroxine (LT4) replacement therapy is the standard regimen for hypothyroidism, aiming to restore normal systemic metabolism and thyroid function. In patients with CSU comorbid with AITD, hypothyroidism is the predominant type of thyroid dysfunction, and subclinical hypothyroidism (SH) is the most common presentation (50). For these patients, standard LT4 treatment not only effectively corrects the hypothyroid state but has also been shown, in multiple clinical cases and observational studies, to alleviate urticarial wheals and pruritus to varying degrees (53, 54), suggesting that LT4 confers additional therapeutic benefit in patients with CSU and AITD (predominantly HT). However, for CSU patients who are euthyroid but positive for anti-TPO antibodies, the clinical benefit and optimal timing of initiating LT4 therapy remain highly controversial. Although studies have shown that anti-TPO antibody levels in these patients are significantly higher than those in healthy populations, indicating potential underlying thyroid autoimmunity (13, 55), there is currently no high-level evidence that LT4 improves their urticarial symptoms; some clinical studies have even suggested a lack of significant benefit (56, 57). Additionally, case reports have shown that CSU symptoms resolved in parallel with declining antibody levels in patients who underwent thyroidectomy, further supporting the involvement of immune-mediated mechanisms in the development and progression of this comorbidity (56). Moreover, although anti-TPO IgE autoantibodies have been detected in patients with CSU, their pathogenicity has not been clearly established (24), which adds to the complexity of treatment strategies for this comorbidity. Based on the above evidence, current data are insufficient to support the routine use of LT4 in CSU patients with normal thyroid function; however, for those with concomitant hypothyroidism, correcting thyroid function is a reasonable adjunctive measure. In clinical management, it is essential to distinguish between thyroid functional status and autoimmune status—the former warrants thyroid hormone replacement, whereas the latter may require immunomodulation—as the two have different implications for guiding treatment decisions. This distinction further underscores that thyroid functional status is a key reference factor in determining the treatment approach.

### 5.2 Targeted therapeutic strategies based on shared immune mechanisms

CSU and AITD frequently coexist, and patients with this comorbidity often present with a more complex clinical picture and significantly greater treatment challenges. The shared pathological mechanisms between the two conditions—including autoimmune injury, activation of inflammatory pathways, MC activation, and complement cascade reactions—provide a theoretical basis for developing biologics and small-molecule drugs that may exert synergistic therapeutic effects. Given the involvement of distinct aberrant immune pathways, targeted therapeutic strategies should be individualized, with full consideration of their specific value and precautions in the context of comorbidity. It should be noted that direct clinical evidence for the following targeted therapeutic strategies in patients with CSU comorbid with AITD remains limited; most recommendations are based on biological plausibility derived from shared immune mechanisms and should be interpreted cautiously in conjunction with evidence from studies of each disease alone (**Table 1**).

**Table 1:** Potential targeted therapeutic strategies and evidence overview for CSU with concomitant AITD.

Target	Potential drugs	Existing evidence (CSU/AITD)	Inferred potential value in comorbidity	Precautions
IgE	Omalizumab	CSU: Standard-dose omalizumab (300 mg every 4 weeks) is supported by high-certainty GRADE evidence; low-dose omalizumab by moderate-certainty GRADE evidence. AITD: No direct clinical evidence.	Patients with type I CSU respond well to omalizumab, whereas those with type IIb CSU may be resistant. Because type IIb CSU is often accompanied by anti-TPO IgG positivity, thyroid antibody status may serve as a reference marker for predicting treatment response.	CSU patients with hypothyroidism and low basophil counts may respond poorly to omalizumab and often require dose adjustment.
IL-4Rα	Dupilumab	CSU: Moderate-certainty evidence (GRADE) for improvement of urticaria activity; no trial data for angioedema; uncertain evidence for quality of life. AITD: No direct clinical evidence.	Theoretically, it may improve GD, which is Th2-dominant, but may exacerbate HT, which is predominantly Th1-mediated.	The Th1/Th2 balance should be assessed individually to avoid exacerbating Th1-dominant HT.
IL-17A	Secukinumab	CSU: Small case series suggest potential efficacy of secukinumab. AITD: No direct clinical evidence; elevated IL-17 levels in both HT and GD suggest a mechanistic association.	It may be effective for CSU refractory to conventional treatment, especially type IIb CSU. IL-17A–targeted therapy is a promising but still experimental immunotherapeutic strategy in AITD.	AITD is driven by a multi-cytokine network; blocking IL-17A alone may induce compensatory activation of other pathways, potentially worsening the disease. Moreover, no unified endpoint currently exists for assessing treatment efficacy.
CD20	Rituximab	CSU: Only case reports suggest that rituximab may be effective. GD: Phase II clinical trials have demonstrated efficacy.	May reduce pathogenic autoantibody production.	The optimal dose, treatment duration, and long-term safety in comorbid conditions require further investigation.
BTK	Remibrutinib	CSU: High-certainty GRADE evidence for reducing urticaria activity and angioedema and improving quality of life; safety evidence is of lower certainty. AITD: No direct clinical evidence; indirect evidence from the BTK inhibitor ibrutinib in thyroid-associated ophthalmopathy (TAO).	May act synergistically by simultaneously interfering with B-cell receptor signaling and mast cell FcϵRI signaling.	No direct evidence in the comorbid population.
JAK-STAT	JAK inhibitors	CSU: Only preliminary small-sample exploratory data are available. AITD: Tofacitinib has been reported for the treatment of TAO.	JAK inhibitors can broadly block signal transduction of multiple cytokine pathways, including Th1, Th2, and Th17.	No direct evidence in the comorbid population.

BTK, Bruton tyrosine kinase; CSU, chronic spontaneous urticaria; FcϵRI, high-affinity IgE receptor; GD, Graves’ disease; GRADE, Grading of Recommendations, Assessment, Development, and Evaluation; HT, Hashimoto thyroiditis; IgE, immunoglobulin E; IL-4Rα, interleukin-4 receptor alpha; IL-17A, interleukin-17A; JAK, Janus kinase; JAK-STAT, Janus kinase–signal transducer and activator of transcription; TAO, thyroid-associated ophthalmopathy; Th, T helper; type IIb CSU, autoimmune endotype of CSU mediated by mast cell-activating autoantibodies (Refences (58–76) are listed in the table).

Accordingly, future research should prioritize clinical studies specifically targeting the comorbid phenotype (**Figure 3**).

**Figure 3:**
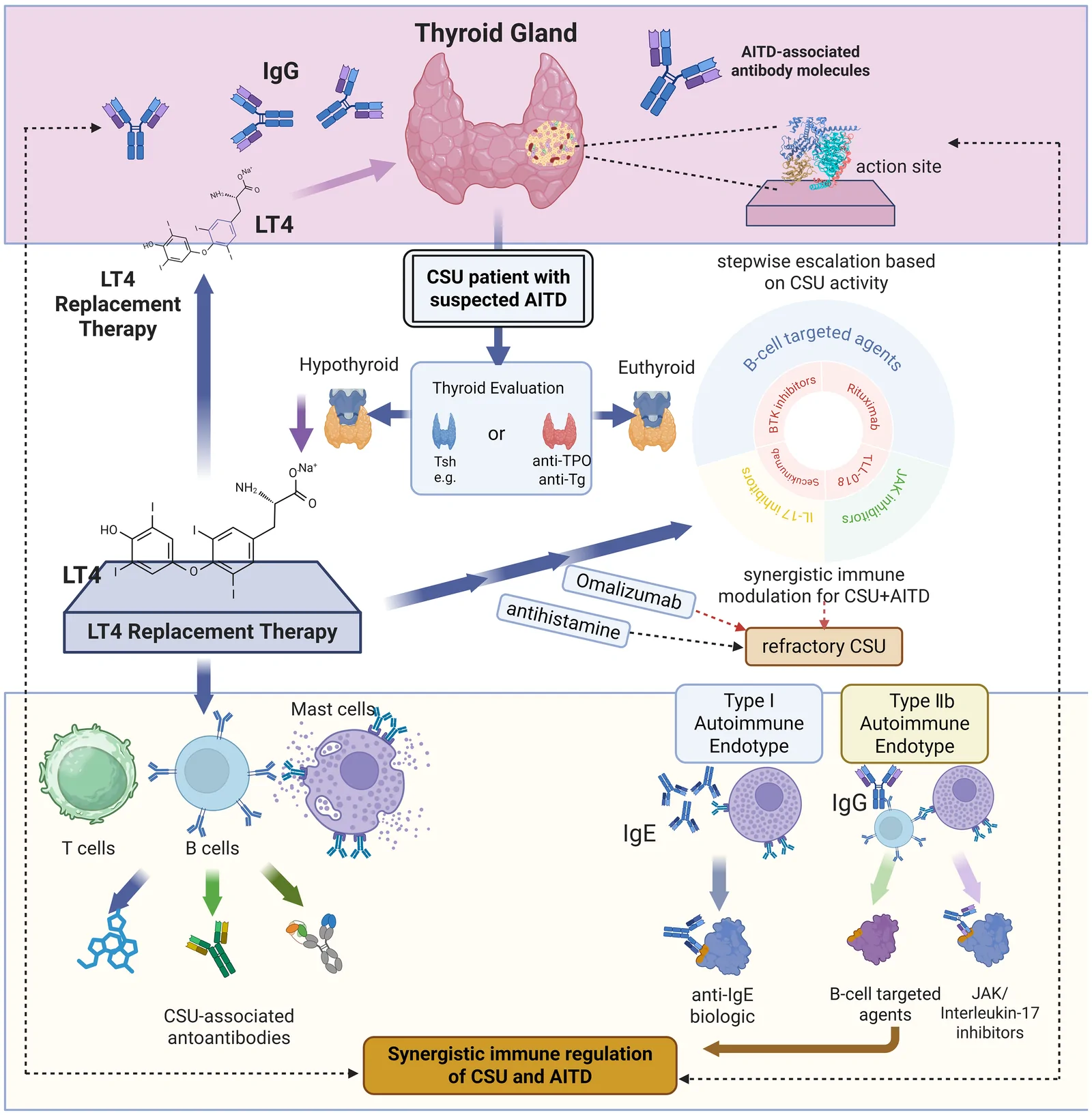
A stepwise, immune-phenotype-guided treatment algorithm for CSU comorbid with AITD. (Created with BioRender.com). The algorithm integrates thyroid evaluation into the standard CSU management pathway. For hypothyroid patients, LT4 replacement is the cornerstone of treatment. For euthyroid patients, treatment escalates based on disease activity. In refractory cases, the classification of autoimmune endotypes (type I vs. type IIb) guides the selection of targeted therapies, aiming for synergistic effects on both CSU and AITD pathophysiology. CSU, chronic spontaneous urticaria; AITD, autoimmune thyroid disease; LT4, levothyroxine.

### 5.3 Establishing a multidisciplinary collaborative management model of “immunology–dermatology–endocrinology”

CSU and AITD are not independent diseases; rather, they represent systemic immune-related comorbidities in which systemic autoimmune dysregulation is one of the key underlying mechanisms, with the skin and thyroid gland as shared target organs (13). They share key pathological mechanisms, including lymphocyte activation, aberrant cytokine signaling, and overlapping autoantibody production, and can influence each other’s disease course and treatment response. If diagnosis and treatment are delivered within a single specialty alone, management is often limited to local symptoms, making it difficult to address immune dysregulation, cutaneous involvement, and endocrine abnormalities as an integrated whole. This may lead to fragmented care and increase the risk of disease recurrence.

For this type of comorbidity, establishing a multidisciplinary team (MDT) involving dermatology, endocrinology, and clinical immunology is worth exploring. This model adopts a systemic immunological perspective, addressing both the cutaneous manifestations of urticaria and thyroid autoimmune abnormalities. Through multidisciplinary joint assessment, the team comprehensively evaluates the patient’s skin condition, thyroid function, and overall immune profile, and collaboratively formulates intervention strategies. This approach integrates symptomatic control of skin lesions with endocrine regulation of thyroid function, facilitates the selection of appropriate therapeutic agents, and avoids medications that may exacerbate thyroid immune injury, with the goal of achieving comprehensive intervention in patients with CSU comorbid with AITD.

It should be noted that the aforementioned “immunology–dermatology–endocrinology” MDT remains a conceptual framework; no prospective studies have yet confirmed its superiority over the traditional single-specialty model of care. Nevertheless, it holds promising potential for further exploration, and high-quality clinical studies are still needed to validate its actual benefits for patients with CSU comorbid with AITD (**Figure 4**).

**Figure 4:**
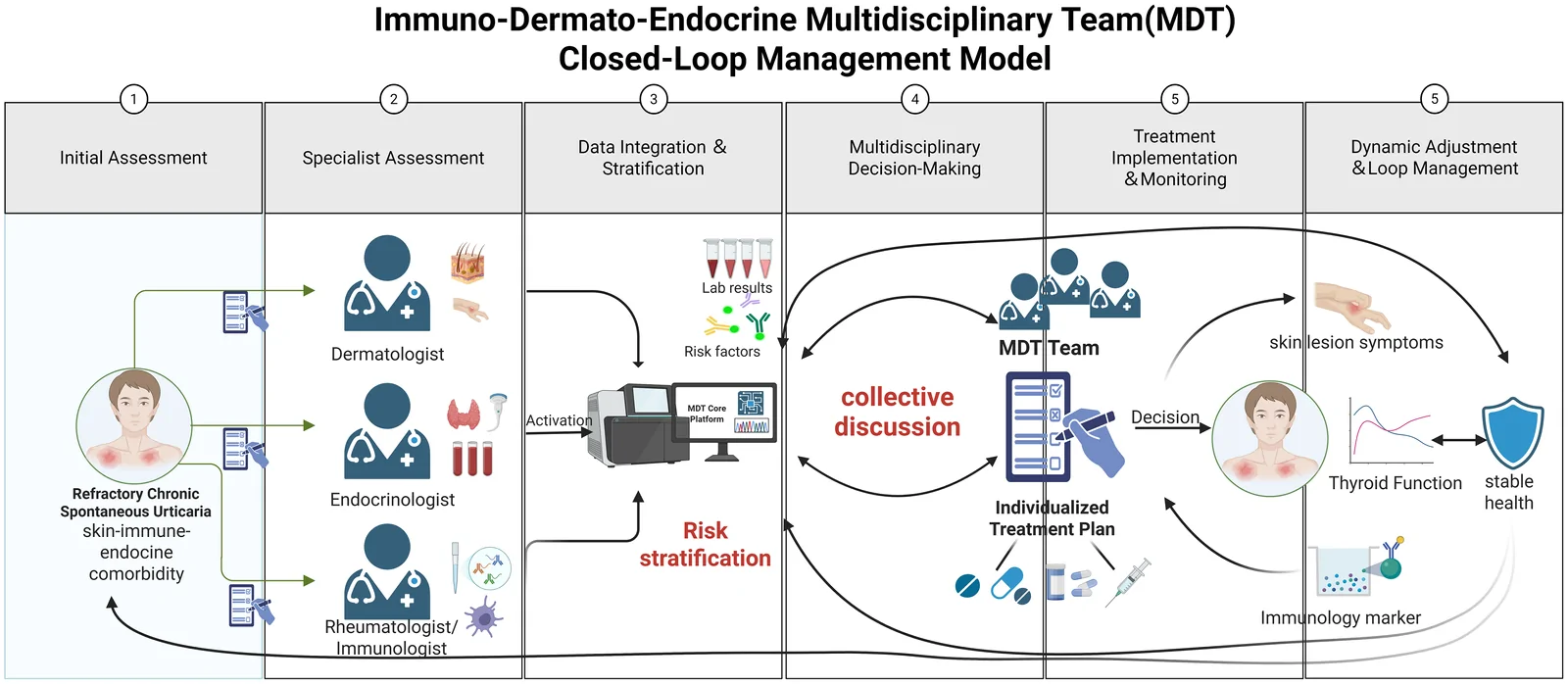
The “immunology–dermatology–endocrinology” MDT management model. (Created with BioRender.com). This figure outlines a closed-loop collaborative management pathway. At the initial evaluation stage, dermatology, endocrinology, and rheumatology/clinical immunology conduct parallel assessments. The MDT core platform integrates all data for risk stratification and shared decision-making. The treatment phase is followed by unified monitoring of skin, thyroid, and immune parameters, allowing dynamic treatment adjustments. This model guides the patient from acute management through stable maintenance, ensuring continuous, integrated care. MDT, multidisciplinary team.

## 6 Conclusions and future perspectives

CSU and AITD frequently coexist, suggesting that the two conditions may share certain immunopathological mechanisms. However, important controversies remain regarding whether thyroid autoantibodies are causative or merely an accompanying phenomenon, and whether correction of thyroid function influences the course of CSU. In clinical practice, it is recommended that thyroid function and thyroid autoantibody screening be incorporated into the baseline assessment of patients with moderate-to-severe or refractory CSU, and that individualized treatment guided by type I/type IIb classification be attempted. However, the current level of evidence for this approach remains low.

Future research should prioritize the following lines of investigation. First, in cohorts that strictly distinguish among normal thyroid function, subclinical hypothyroidism, clinical hypothyroidism, and euthyroid antibody-positive status, it should be evaluated whether levothyroxine treatment can improve urticarial symptoms. Second, the predictive value of the combination of low serum total IgE, anti-TPO IgG positivity, and a positive ASST for type IIb classification and targeted therapy response should be validated. Third, randomized controlled trials should be conducted in comorbid populations to compare the efficacy and safety of bruton tyrosine kinase (BTK) inhibitors with relatively mature evidence (e.g., remibrutinib) and of Janus kinase (JAK) inhibitors, which are still at an early exploratory stage, with that of omalizumab in patients with type IIb refractory CSU. The “immunology–dermatology–endocrinology” integrated multidisciplinary management model remains a conceptual framework, and whether it can improve outcomes in patients with CSU–AITD comorbidity awaits confirmation by prospective studies.

## Glossary

3,5-T2: 3,5-diiodothyronine
AITD: Autoimmune thyroid disease
anti-TG/anti-Tg: Anti-thyroglobulin antibodies
anti-TPO: Anti-thyroid peroxidase antibodies
ASST: Autologous serum skin test
BTK: Bruton’s tyrosine kinase
C1q: Complement component 1q
C3: Complement component 3
C4: Complement component 4
C4d: Complement component 4d
C5a: Complement component 5a
CD20: B-lymphocyte antigen CD20
CSU: Chronic spontaneous urticarial
FcϵRI: High-affinity Fcϵ
FT3: Free T3
FT4: Free T4
GD: Graves’
HR: Hazard ratio
HT: Hashimoto’s thyroiditis
IFN-γ: Interferon-gamma
IFN-λ1: Interferon-lambda 1
IgE: Immunoglobulin E
IgG: Immunoglobulin G
IL-4: Interleukin-4
IL-4Rα: interleukin-4 receptor alpha
IL-6: Interleukin-6
IL-10: Interleukin-10
IL-17: Interleukin-17
IL-21: Interleukin-21
IL-23: Interleukin-23
IL-24: Interleukin-24
IL-29: Interleukin-29
IL-35: Interleukin-35
JAK: Janus kinase
JAK-STAT: Janus kinase–signal transducer and activator of transcription
LT4: Levothyroxine
MDT: Multidisciplinary team
SH: Subclinical hypothyroidism
T1AM: 3-iodothyronamine
TA1: 3-iodothyroacetic acid
Th1: T helper type 1
Th2: T helper type 2
Th17: T helper type 17
TPO: Thyroid peroxidase
TRAb: Thyrotropin receptor antibodies
TSAb: Thyroid-stimulating antibodies
TSH: Thyroid-stimulating hormone
